# Age-Related Differences in Vehicle Control and Eye Movement Patterns at Intersections: Older and Middle-Aged Drivers

**DOI:** 10.1371/journal.pone.0164124

**Published:** 2016-10-13

**Authors:** Yusuke Yamani, William J. Horrey, Yulan Liang, Donald L. Fisher

**Affiliations:** 1 Department of Psychology, Old Dominion University, Norfolk, VA, United States of America; 2 Center for Behavioral Science, Liberty Mutual Research Institute for Safety, Hopkinton, MA, United States of America; 3 Department of Industrial and Mechanical Engineering, University of Massachusetts, Amherst, MA, United States of America; 4 Volpe National Transportation Center, Cambridge, MA, United States of America; University of Nottingham, UNITED KINGDOM

## Abstract

Older drivers are at increased risk of intersection crashes. Previous work found that older drivers execute less frequent glances for detecting potential threats at intersections than middle-aged drivers. Yet, earlier work has also shown that an active training program doubled the frequency of these glances among older drivers, suggesting that these effects are not necessarily due to age-related functional declines. In light of findings, the current study sought to explore the ability of older drivers to coordinate their head and eye movements while simultaneously steering the vehicle as well as their glance behavior at intersections. In a driving simulator, older (M = 76 yrs) and middle-aged (M = 58 yrs) drivers completed different driving tasks: (1) travelling straight on a highway while scanning for peripheral information (a visual search task) and (2) navigating intersections with areas potential hazard. The results replicate that the older drivers did not execute glances for potential threats to the sides when turning at intersections as frequently as the middle-aged drivers. Furthermore, the results demonstrate costs of performing two concurrent tasks, highway driving and visual search task on the side displays: the older drivers performed more poorly on the visual search task and needed to correct their steering positions more compared to the middle-aged counterparts. The findings are consistent with the predictions and discussed in terms of a decoupling hypothesis, providing an account for the effects of the active training program.

## Introduction

The number and proportion of older adults are on the rise and the number of older drivers is expected to increase dramatically in developed countries including the United States. This leads to a looming problem: the fatal vehicle crash rate per mile begins to increase at age 70, dramatically rising after age 75; drivers 85 and older are 8.8 times more likely to be involved in a motor vehicle crash than drivers aged 40–44 [1]. Moreover, when involved in crashes, older adults are more likely to be severely injured or die because of greater frailty [2, 3]. Among all of the crash types, adults aged 70 and older face a much greater risk of crashing at intersections [1, 4]. Specifically, multi-vehicle fatal crashes at intersections accounted for roughly 35% of total crash involvements among drivers 80 and older while only 18% among drivers between 20 and 59 [1]. Several studies further indicate that left turns at a T-intersection or across traffic, in particular, pose a greater risk for older adults [5–7]. Perhaps not surprisingly, older adults report difficulty in maneuvering at intersections [8] and tend to show poorer decision skills at intersections compared with younger, experienced drivers [9].

Given the inherent complexity of intersections, drivers must actively scan many different sources of information including other vehicles and pedestrians in order to maneuver intersections successfully. Due to the dynamic nature of the driving environment, information sources often must be re-scanned several times in order to reduce uncertainty regarding the state of the world [10]. Successful intersection maneuvers require the ability to perceive hazards in such a dynamic driving environment, and patterns of eye movements during driving can reflect the ability to anticipate latent road hazards for novice drivers [11, 12] and older drivers [13]. For example, Pradhan and colleagues [11] asked novice drivers, young drivers, and older drivers to drive in various scenarios each of which contained a latent hazard, a hazard which has not yet materialized, in a high-fidelity driving simulator and measured their eye movements. The results indicate that novice drivers failed to fixate the hazard, and thus acquire information about its risk, while the other groups did not.

Previous works also suggest that older drivers maintain their ability to scan a traffic scene and detect hazards while driving [14, 15]. For example, Borowsky and colleagues [14] asked young, middle-aged experienced and older drivers to detect hazard while viewing movies containing several materializing and latent hazard. Older drivers detected materializing and latent hazard not only as equally proficiently as middle-aged drivers but also significantly better than younger drivers following a hazardous event. Eye movement data in Reimer et al. [15] study further indicate that older drivers’ gaze concentration, a variability of gaze positions, was no different than that of younger drivers, suggesting the sustained ability to actively scan driving environments among older drivers. However, studies focusing on intersection behaviors have accumulated evidence for age-related differences in visual scanning patterns while negotiating various intersections: older adults are less likely to scan outside of the intended path of the vehicle before executing a turn through intersections, compared with young and middle-aged adults both in the field [5] and in driving simulators [13, 16, 17].

**Fig 1:**
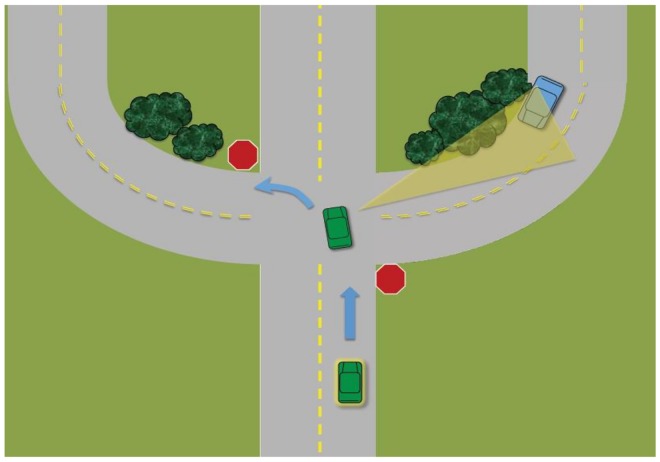
A schematic example of a sign-controlled 4-way intersection with a target zone (area shaded by yellow cone) that contains a latent hazard. (Prior to the stop sign, drivers should make primary glances side to side. After entering the intersection and before executing a turn, drivers should take secondary glances to the areas defined as the target zones. In this example, drivers should look toward the target zone before making a left turn. The blue car—a latent hazard—was not actually present in the scenario).

Various studies have differentiated between primary and secondary glances at intersections [16, 18]. Primary glances are defined as visual scanning to either side when drivers are approaching or stopped at an intersection and waiting for a break in traffic. Secondary glances are defined similarly, but occur after drivers have begun accelerating into the intersection (for example, see Fig 1). Secondary glances are often necessary at intersections because the road geometry or the built or natural environment can obscure cross-traffic, possibly masking potential hazards. Primary and secondary glance patterns differ between older and middle-aged drivers. In a simulator study by Romoser and Fisher [16], for example, older adults were half as likely to execute secondary glances as middle-aged drivers. Furthermore, proportion of correct secondary glances was negatively associated with observed crashes in driving simulator for older drivers but not for middle-aged drivers [18], indicating criticality of secondary glances at intersections.

It remains, however, unclear why older adults exhibit different secondary glance patterns from middle-aged drivers and what mechanisms underlie older adults’ allocation of attention more heavily in front of their vehicle than to the sides, where potential threats often occur when negotiating intersections (See [19] for review]. In general, it is well-documented that normal aging is accompanied by changes in perceptual-cognitive-motor abilities [20, 21]—changes that can impact driving behavior and possibly lead to a crash. More specifically, one can identify several mechanisms that may account for older drivers’ failure to properly scan at intersections, such as increased difficulties with: (a) large head movements [22], (b) psychomotor coordination [23], (c) multitasking [24], (d) tasks which draw heavily on working memory [25, 26], (e) distractions [27], (f) scanning complex displays [26], (g) restricted useful field of view [28], and (h) vision [29].

While important deficits in their own right, these explanations cannot by themselves fully account for observations by Romoser and Fisher [16], who found that older drivers’ secondary glances at intersections in the field could be doubled by a short simulator training program. In the program, they gave older drivers practice on the simulator navigating intersections and taking secondary glances to the sides. Critically, drivers in the active learning group showed a dramatic increase in the frequency of secondary glances observed in the field three months after training, from roughly 40% pre-training to 80% post-training, whereas neither the passive nor control group showed any changes or benefits. A recent follow-up study of the trained drivers indicates that the effectiveness of the active training persisted two years following the original training [30], suggesting that older drivers do not return to their “bad habit” if they have received the training program.

The training program did not however explicitly target any of the above cognitive, perceptual, or motor components that could decline with normal aging per se. One possible explanation for the effectiveness of the training program over the short and long term is that, before training, older drivers treated intersection navigation as a single-phase maneuver. That is, older drivers attempted to move their head and eyes along with their hands and feet simultaneously—as would younger drivers; however, the increased, perhaps excessive, demand of multi-limb and eye/head coordination led to neglect of the secondary glances, perhaps due to age-related decrease in attentional resources [20]. With simulator training [16], however, older adults were able to apportion the tasks and manual inputs required for intersection navigation into two phases. First, the driver slowly accelerated, straight into the intersection (minor pedal [foot] inputs and limited steering [hand] inputs), allowing for glances to the side, away from the path of travel (eye/head movements). Second, having executed the secondary glances, the driver initiated the turn (larger foot and hand inputs). Thus, this two-phase maneuver could increase the frequency of secondary glances for older drivers. The notion that older drivers try to reduce the number of simultaneous movements of their extremities is consistent with other simulator studies of intersection navigation [31].

The current study aims to examine, in the same group of drivers, secondary glance behavior at intersections and their coordination of vehicle control and lateral scanning in the same group of drivers. As noted, the latter task could shed some insight into the behavioral and performance costs associated with coupling off-center eye/head movements with vehicle control. Specifically, the study seeks to (1) replicate the effects of aging on secondary glance behavior at intersections in a different simulator platform from Romoser and Fisher [16] and (2) examine whether the same drivers are able to successfully coordinate steering inputs (i.e., maintain the heading of the vehicle) while gathering information from visual displays, presented on the side of the road. If older drivers fail to execute secondary glances due to their difficulty to execute concurrent multilimb responses at intersections, they should also exhibit difficulty in performing a task that requires visual scans away from road center while controlling a vehicle in a driving environment with wind turbulence. To test this prediction, both middle-aged and older drivers completed two different tasks in a driving simulator.

- *Task 1*: *Intersections*. To address Aim 1, participants were asked to navigate eight intersection scenarios that require the driver to scan to the sides as he or she approaches and enters the intersection in order to look for potential hazards (i.e., which require secondary glances). This condition was intended to replicate the scenarios used by Romoser & Fisher [16]. We predicted that older drivers would execute a smaller proportion of correct secondary glances than middle-aged drivers.
- *Task 2*: *Visual Search*. To address Aim 2, participants were asked to perform a concurrent visual search task located in a fixed position to the left or right hand side of the road, and designed to direct the participants’ eyes and head away from the forward roadway. In order to also examine the differential impact of increased driving (control) difficulty, they performed the task in three different conditions: while driving with or without wind turbulence and in static baseline conditions (no driving). We predicted that older drivers would show poorer visual search performance and vehicle control than middle-aged drivers. Furthermore, the presence of wind turbulence is hypothesized to increase driver workload and thus decrease visual search performance [15]. With respect to the baseline condition, we predicted that older and middle-aged drivers would demonstrate comparable levels of search performance.

## Methods

### Subjects

Twelve middle-aged (5 females, mean age = 58.3 years old, SD = 4.75 years, range = 50–65, mean far acuity = 20/21.0; mean maximum head rotation = 59.3±12.0°) and twelve older (9 females, mean age = 75.7 years old, SD = 4.11 years, range = 70–85, mean far acuity = 20/26.7; maximum head rotation = 55.3±10.1°) drivers participated in the study. Three middle-aged and 7 older subjects wore glasses during the experiment. All subjects held a valid driver’s license and drove, on average, 18,500 km per year (M = 21,500 km vs. 15,700 km for middle-aged and older drivers, respectively). This study was reviewed and approved by UMass IRB #2013–1817.

### Apparatus

A medium-fidelity simulator (Systems Technology Inc., Hawthorne, CA) with three 60″ projection screens, subtending 160 degrees of the viewing angle from the driver’s seat, and a head-mounted eye tracker (Applied Science Laboratory, Bedford, MA) with the sampling rate of 30 Hz, were used for the study. The participants viewed the center and side screens from the distance of approximately 170 cm. The eye tracker is estimated to have 0.5 degrees tracking accuracy in visual angle and was calibrated using a 9 point calibration grid.

### Scenarios and Search Tasks

As noted, the experiment consisted of two tasks–intersection (Task 1) and visual search scenarios (Task 2). Task 2 involved two blocks with concurrent driving and visual search and one block involving the visual search task alone.

#### Task 1: Intersections

Participants drove a series of eight scenarios similar to those used in Romoser and Fisher [16] in a random order. Two of the scenarios did not contain latent hazards in order to minimize subjects’ expectation of such events. The other six intersection scenarios included various latent hazards that could appear from the side, outside of the driver’s field of view, thus requiring a head and eye movement to confirm that no hazard was present (see Table 1 for a description of the scenarios). Fig 1 illustrates a stop sign-controlled intersection scenario where participants needed to turn left onto the appropriate lane while monitoring for oncoming traffic from the right side. For this lane, the line of sight to the right is restricted by the presence of roadside shrubbery, thus requiring that drivers closely monitor the region to avoid a potential collision during the turn. Scenario 3 contained a vehicle on the opposing lane approaching the participant’s vehicle, and Scenario 4 involved cross traffic present when the scenario began. In both cases, the vehicles did not create additional target zones nor mask any predefined target zones. No ambient traffic was present in the remaining scenarios.

**Table 1:** Intersection scenarios and the corresponding location of target zones.

	Scenario	Description
1	Left Turn at Divided Highway with Median (Rural)	Signalized intersection with two lanes in each direction. Participant’s Vehicle (PV) makes left turn on green. Downward vertical curvature (hill) obscures the opposing lane. Subjects are expected to make a secondary glance towards the curvature.
2	Left Turn at Divided Highway with Median (Urban)	Signalized intersection with two lanes in each direction. PV makes left turn on green. Horizontal curvature and an office building obscure the opposing lane. Subjects are expected to make a secondary glance toward the curvature.
3	Left Turn at Intersection Across Traffic (Rural)	Stop sign at intersection with cross-traffic. PV makes left turn after a full stop. Crossroad is two lanes, one in each direction. Horizontal curvature and bush obscure cross-traffic. While making a left turn, subjects are expected to confirm no cross-traffic is present (see Fig 1).
4	Left Turn at Intersection Across Traffic (Urban)	Stop sign at intersection with cross-traffic. PV makes left turn after a full stop and a truck passing the intersection on the opposing lane. Crossroad is two lanes, one in each direction. Horizontal curvature and office buildings obscure cross-traffic. While making a left turn, subjects are expected to confirm no cross-traffic is present.
5	Left Turn at T intersection	Stop sign at T intersection. PV makes left turn after a full stop. Bush to the right. Subjects are expected to confirm no cross-traffic is present before they turn.
6	Right Turn at T intersection	Stop sign at T intersection. PV makes right turn after a full stop. Parked trucks on the curb to the left. Subjects are expected to confirm no cross-traffic is present before they turn.

#### Task 2: Visual Search

Participants performed a visual search task with and without a concurrent driving task. For the baseline (static) visual search task, drivers were seated in the cab of the automobile and asked to perform the visual search task while remaining stationary (i.e., no driving took place). An experimenter controlled the onset of the visual search display for each trial with an inter-stimulus interval of approximately 10 seconds.

In the driving blocks, participants were asked to drive a two-lane highway in a rural environment with sparse vegetation on the sides and occasional horizontal road curvature. There was no ambient traffic present and the posted speed limit was 35 mph. Two forms of this scenario were used: one with and one without lateral wind turbulence, constituting two experimental blocks. The lateral force of the wind turbulence was generated with a combination of three simple sinusoidal waves (1, 3, and 6 Hz) with the lateral wind speed of 1 foot/second.

**Fig 2:**
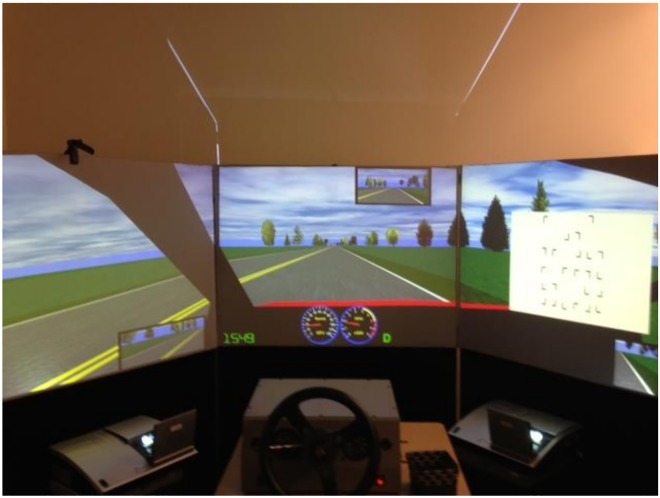
A sample display of highway scenarios in a rural environment with the concurrent search task.

For both driving and non-driving search blocks, drivers performed a concurrent visual search on displays that occasionally appear randomly on either the right or left screen. Fig 2 illustrates the road environment and the placement of the visual search display. The search task involved target and distractor items known to produce inefficient search (cf. 75 ms/item; Wolfe, 1994). Each search display was approximately 30 degrees high and 39 degrees wide and contained 24 items, each subtending approximately 2.3 degrees of visual angle. The items were randomly rotated and located, drawn on black [RGB = 0, 0, 0] on the white background [RGB = 255, 255, 255]. The task was to detect the target, T, among distractors, Ls. To reduce variability of total search times, 80% of the search displays were target-absent trials. Each search display was projected at 30 degrees to the left or right from the point of expansion on the central display to the center of the search display and remained visible for 2 seconds. The search task was intended to direct drivers’ eyes or head away from the road ahead. For the driving block with wind, the direction of wind turbulence was independent of the location of the visual search display.

For each driving block (with and without wind), subjects performed 20 trials of the visual search task with an inter-stimulus interval of approximately 9.7 seconds evenly spread throughout each drive.

### Procedure

At the start of the 90-minute session, participants completed the informed consent as well as a demographic questionnaire. Participants provided their written informed consent to participate in the study. Participants then completed practice trials for the intersection scenarios (left and right turns) and the visual search task. Following practice, participants completed 4 experimental blocks (1 intersections; 3 visual search: low wind, high wind, baseline). The order of the four blocks was counterbalanced across participants and the order of eight scenarios within the intersection block (Task 1) was randomized.

For Task 1, subjects were instructed to navigate through a series of intersections as they would do in the natural environments while obeying the traffic rules (e.g., full stop at a stop sign). Each scenario lasted approximately 10 seconds. For Task 2, participants were asked to provide a verbal response (target present or absent) as soon as they detected the target within each search display, and the experimenter recorded it for each trial. For blocks involving driving (both wind and no wind), participants were instructed to obey the traffic rules (e.g., keep to the speed limit of 35 mph). Each drive took approximately 5 minutes. After completing the experimental blocks, participants were debriefed and remunerated.

### Dependent variables and analysis

#### Task 1: Intersections

The dependent variable was the proportion of intersection scenarios where correct primary and secondary glances were taken (of the six total intersection scenarios). The *target zones* were defined as the areas where potential latent hazards could be present and were specific to each scenario (see Table 1 for detail). Two experienced raters, blind to the age of the participants, independently coded whether each participant executed a secondary glance (1 for correct glance, 0 otherwise) towards the target zones prior to exiting the crossover of an intersection. The difference between primary and secondary glances lies in the early and late launch zones while the target zone remains the same. Primary glances were affirmed if the eye point fell within the target zone when the participant approached the intersection, beginning about 30 feet in advance of the stop line/sign. Secondary glances were similarly affirmed and were considered following the start of the execution of the intersection maneuver (i.e., after an accelerator press was recorded in the simulator data stream). This allowed coders to distinguish secondary from primary glances, per the definition by Romoser & Fisher [16]. Disagreements were resolved through discussion; five observations (6.9% of the data) could not be classified and were excluded from the analysis. The remaining data were submitted to an independent-samples t-test.

#### Task 2: Visual Search

For the visual search task, dependent variables included the presence of at least one glance at the visual search display, the total glance time on each search display (up to 2 seconds, the maximum time the display was visible), and the mean target detection rates based on subjects' verbal reports in the target-present trials. The data were analyzed only for those participants with valid eye data in at least 5 trials. For the analysis, the data were submitted to separate mixed-model ANOVAs with age (middle-aged vs. older) as a between-subject factor and wind (no-wind vs. wind) as a within-subject factor.

With respect to driving performance, the dependent variables included the mean lateral position variability (in ft.) and mean steering input variability (in degrees) as well as mean lateral position of the vehicle (in ft., from the center of the lane). The performance measures were calculated separately for sections of the roadway with or without the search task. The data were then submitted to separate mixed-model analyses of variance (ANOVAs) with age (middle-aged vs. older) as a between-subject factor and wind (no wind vs. wind) and search display (absent, right display, vs. left display) as within-subject factors.

For the baseline visual search block, the proportion correct detections in the search task was the dependent variable. The data were submitted to an independent-samples t-test.

## Results

### Task 1: Intersections

#### Proportion of primary glances

Consistent with the previous report [18], older drivers made statistically significantly smaller proportion of correct primary glances than middle-aged drivers [M = 70% vs. 94%; *t* (22) = 3.04, *p* < .01].

#### Proportion of secondary glances

Consistent with the previous reports [16, 18], older drivers executed statistically significantly fewer secondary glances than middle-aged drivers [M = 31% vs. 64%; *t* (22) = 2.78, *p* = .01]. Moreover, the percentage of secondary glances of the older drivers was roughly half that of the middle-aged drivers.

### Task 2: Visual Search

#### Driving Performance

**Fig 3:**
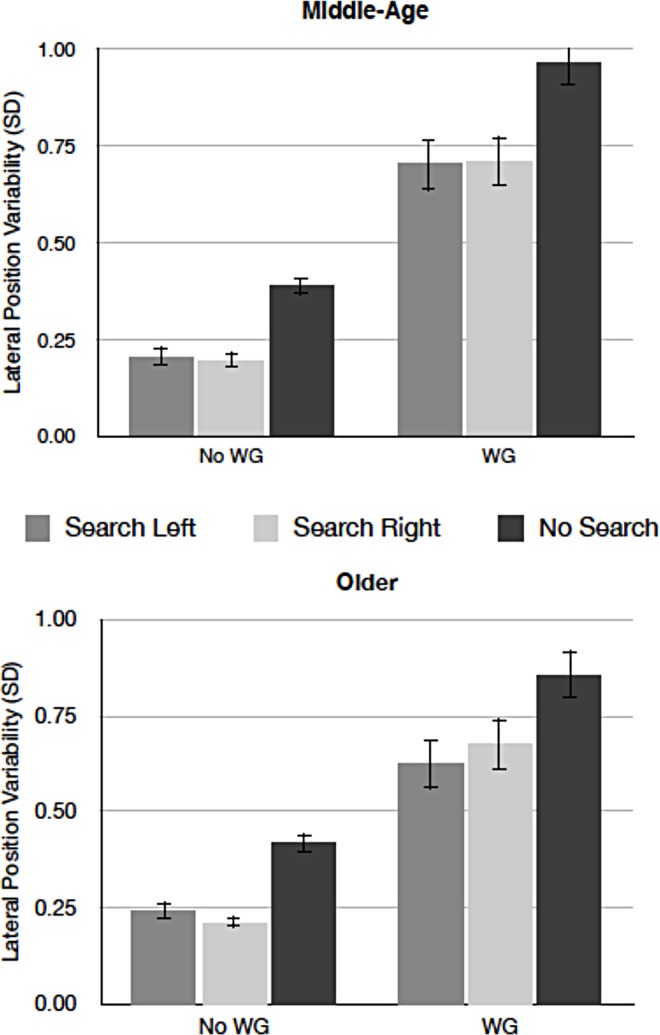
Mean lateral position variability as a function of search display and wind for middle-aged (top) and older drivers (bottom). (WG = wind gusts present, No WG = no wind gust present. Error bars represent standard errors of the means).

*Lateral position variability*. Fig 3 presents mean lateral position variability as a function of search display and wind for middle-aged and older drivers. Variability of lateral position was greater when the wind turbulence was present than absent [*F* (1,22) = 155.42, *p* < .01, *MSE =* .053, *η*^*2*^_*p*_ = .88]. The presence of search displays reliably reduced the variability [*F* (1,22) = 83.42, *p* < .01, *MSE =* .008, *η*^*2*^_*p*_ = .74]. The variability was similar between middle-aged and older drivers [*F* < 1, n.s.], and the remaining interaction effects were not significant [all *p*s > .20].

**Fig 4:**
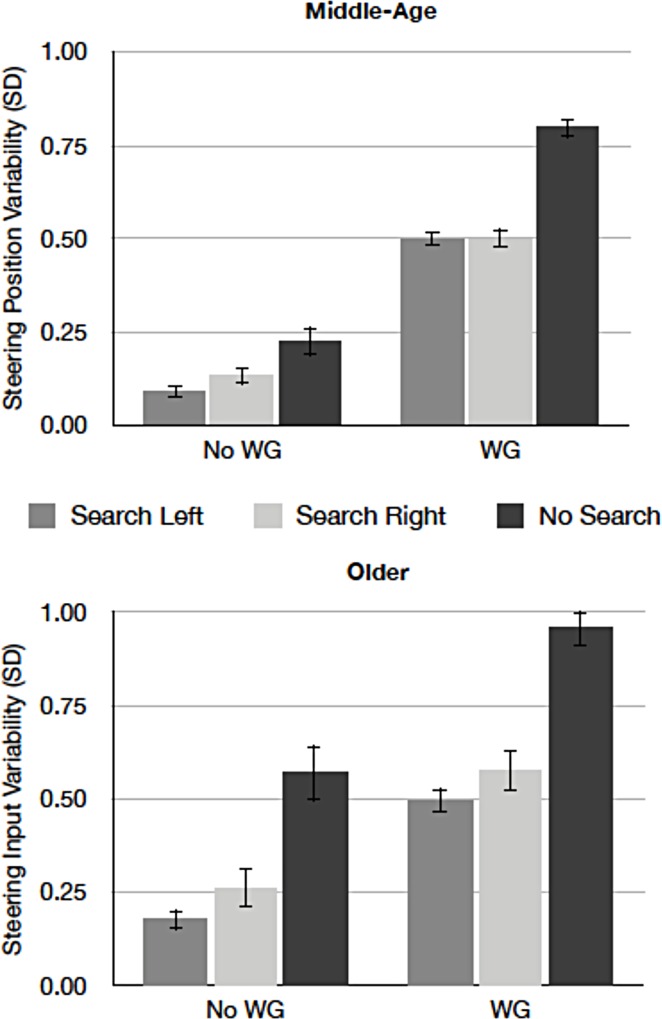
Mean steering input variability as a function of search display and wind for middle-aged (top) and older drivers (bottom). (WG = wind gusts present, No WG = no wind gust present. Error bars represent standard errors of the means).

*Steering input variability*. Fig 4 presents mean steering input variability as a function of search display and wind for middle-aged and older drivers. Older drivers’ steering input was more variable than middle-aged drivers’ [*F* (1, 22) = 9.30, *p* < .01, *MSE =* .066, *η*^*2*^_*p*_ = .30]. Drivers made more variable steering inputs when the wind turbulence was present than absent [*F*(1, 22) = 472.97, *p* < .01, *MSE* = .012, *η*^*2*^_*p*_ = .96], and this difference was more pronounced for older than middle-aged drivers [*F* (1, 22) = 9.49, *p* < .01, *MSE =* .053, *η*^*2*^_*p*_ = .30]. The presence of the search task reduced the variability [*F* (2, 44) = 128.68, *p* < .01, *MSE =* .011, *η*^*2*^_*p*_ = .85], a difference greater in the wind than the no wind condition [*F* (2,44) = 16.04, *p* < .01, *MSE =* .004, *η*^*2*^_*p*_ = .42] and for older than middle-aged drivers [*F* (2, 44) = 12.73, *p* < .01, *MSE =* .011, *η*^*2*^_*p*_ = .37]. The three-way interaction reached statistical significance [*F* (2, 44) = 3.42, *p =* .*04*, *MSE =* .004, *η*^*2*^_*p*_ = .13]. To explore this interaction, two ANOVAs with wind and search display as within-subject factors were run separately for the middle-aged and the older groups. For the middle-aged drivers, the presence of wind increased steering variability [*F* (1, 11) = 371.79, *p* < .01, *MSE =* .010, *η*^*2*^_*p*_ = .97]. The presence of search displays reduced steering input [*F* (1, 22) = 72.89, *p* < .01, *MSE =* .005, *η*^*2*^_*p*_ = .86], but this pattern arose only when wind turbulence was present [*F* (2, 22) = 25.24, *p* < .01, *MSE =* .003, *η*^*2*^_*p*_ = .69]. For the older drivers, again, the steering variability was greater when wind turbulence was present than absent [*F* (1, 11) = 148.79, *p* < .01, *MSE =* .014, *η*^*2*^_*p*_ = .93]. The older drivers also significantly reduced steering variability when concurrently performing the search task on the sides [*F* (2, 22) = 70.12, *p* < .01, *MSE =* .017, *η*^*2*^_*p*_ = .86]; yet, this pattern appeared regardless of the presence of wind turbulence [*F* (2, 22) = 1.81, *p* = .18, *MSE =* .006, *η*^*2*^_*p*_ = .14], indicating that the older drivers reduce their inputs even when the driving environment does not require additional demand for vehicle control.

**Fig 5:**
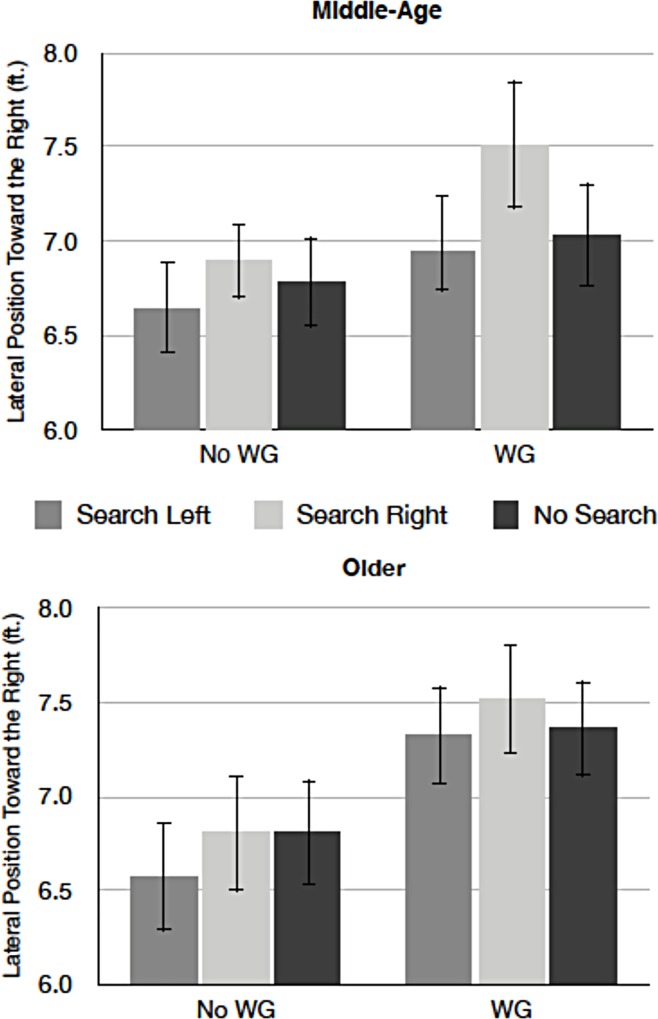
Mean lateral vehicle position as a function of display and wind for middle-aged (top) and older drivers (bottom). (WG = wind gusts present, No WG = no wind gust present. Error bars represent standard errors of the means).

*Lateral vehicle position*. Fig 5 presents mean lateral vehicle position as a function of search display and wind for middle-aged and older drivers. Middle-aged drivers drove closer to the side of the road where the search display was presented, compared to when the search display was absent [*F* (2, 44) = 14.86, *p* < .01, *MSE =* .08, *η*^*2*^_*p*_ = .40]. In general, drivers drove more towards the right side of the lane when the wind turbulence was present than absent [*F* (2, 44) = 21.91, *p* < .01, *MSE =* .46, *η*^*2*^_*p*_ = .50]. The remaining effects were not reliable [all *p*s > .11].

#### Visual Search Performance

*Proportion of glances to the side*. Middle-aged and older drivers glanced at the search displays equally frequently [M = .97 vs. .98, respectively; *t* < 1, n.s.].

*Total glance time toward the search display*. Older drivers tended to spend less time glancing at the search display than middle-aged drivers, but the difference was not statistically reliable [M = 1.15 vs. 1.53 sec, *F* (1, 22) = 2.07, *p* = .17, *MSE* = 534.13, *η*^*2*^_*p*_ = .12]. Mean total glance time was numerically shorter when the wind turbulence was present than absent, although this effect was not significant [M = 1.27 vs. 1.42, *F* (1,22) = 4.17, *p* = .059, *MSE* = 40.42, *η*^*2*^_*p*_ = .22]. The interaction effect was not reliable [*F* < 1, n.s.].

*Target detection rates in the visual search task*. In the driving scenarios (Task 2), mean target detection rates were lower for older drivers than middle-aged drivers [M = 0.58 vs. 0.78; *F* (1,22) = 6.77, *p* = 0.02, *MSE =* 0.07, *η*^*2*^_*p*_ = 0.24]. The presence of wind turbulence numerically decreased the detection rates, but the main and interaction effects were not reliable [both *p*s > 0.55]. False alarm rates were low for both middle-aged and older drivers (0 vs. 0.008, n.s.).

*Baseline (Static) Proportion correct detections in the visual search task*. When performing the visual search task only, mean proportion correct detections were not statistically significantly different between older and middle-aged drivers [M = 0.79 vs. 0.91, *t* (22) = 1.12, *p* = 0.28].

## Discussion

Older drivers (70+) face an elevated risk of a fatal vehicle crash at intersections [1], even though previous works suggest that their hazard anticipation ability sustains across normal aging [14,15]. In part, this is perhaps because older drivers often fail to execute secondary glances for potential hazards at intersections [16]. While the current and previous works have found that older drivers scan less frequently to the periphery at intersections in general [5, 17], secondary glances are considered critical as they have been linked to crash involvement in simulator studies [18]. This study replicated prior results, with older drivers taking secondary glances at intersections only half as often as middle-aged drivers. We further extended these earlier works by examining vehicle control in these same drivers in the context of a peripheral visual search task. Such tasks required a head/eye movement away from the forward roadway and thus demanded the ability to couple multiple motor responses while driving. Importantly, we note that the two tasks used in the current study were not intended to allow for inferences about how people scan in one circumstance (i.e., driving on a straight highway) compared to another (i.e., navigating an intersection). Rather, the interest lies in the ability of older drivers to control their vehicle while glancing to the periphery (i.e., the coupling of eye/head movements and steering inputs). This is associated with what has been referred to elsewhere as the decoupling hypothesis [32]; if older drivers decouple the turning and accelerating functions from the glances to the side, they navigate intersections with increased frequency of secondary glances, enhancing their safety [31]. In the current context, while traveling straight in the visual search task, older drivers should fail to glance to the side, leading to poor visual search performance, in order to maintain the position of the vehicle, or vice versa, and this pattern may be exacerbated by increased task demand imposed by heavy wind turbulence.

The current results were consistent with the predictions of the decoupling hypothesis that older drivers *couple* multi-limb responses independent of the active training program by Romoser and Fisher (2009), neglecting secondary glances at intersections from the chain of motor responses necessary for safe intersection maneuvers. First, in Task 1 (Intersections), the present study replicated the age-related loss of secondary glances at intersections. Second, in the same group of older drivers in Task 2 (Visual Search), they performed the visual search task comparably well compared to the middle-aged drivers when they were asked to perform the task alone. However, although the older drivers were able to glance towards the search displays while driving, they performed the visual search task more poorly than the middle-aged drivers. Third, the older drivers required more steering corrections in order to maintain the vehicle position to the level comparable to that of the middle-aged drivers when concurrently performing the peripheral visual search task. These data point to the tendency to ‘couple’ multi-limb responses while driving for older drivers, reflecting their compromised ability to multitask when the tasks require concurrent execution of multiple responses—an outcome which may lead to the neglect of secondary glances found in the current study and previous works.

Importantly, the current results also carry implications for the results from Romoser, both the results three months after training [16] and two years after training [30]. First, consider the results three months after training. They found that three months after simulator training the older drivers doubled the frequency of secondary glances when measured in the field. This is what would be predicted if the older drivers treated intersection navigation as a single-phase maneuver before training on the driving simulator and a two-phase maneuver after training, and if the older drivers could not coordinate secondary glances with turning and accelerating when the intersection navigation was treated as a single phase. When treated as a two-phase maneuver, older drivers can turn their head, maintain lane position, and gather information successfully from the sides during the straight phase. The most compelling reason why they cannot do such during the turning phase is the difficulty in coordinating head and eye movements with hand and foot movements. All that said, we can only speculate on the implications of the training program based on the current results; future research should directly evaluate the hypothesis that older drivers change from navigating the intersection as a one-phase maneuver rather than a two-phase maneuver, after completing the active training program.

Second, consider the results two years after training [29]. Although a slight decrease in secondary glances among the actively trained group was observed, it was not statistically significant. Instead of the other age-related visual/perceptual/cognitive deficits, the current results implicate the decoupling of multiple motor responses in the active training program for older drivers, allowing them to regain the ability to make secondary glances. The current results indicate that the steering position variability during the straight sections both with and without wind was greater for the older drivers than the middle-aged drivers, almost by a factor of two. This indicates that, on the driving simulator, older drivers are consistently oversteering and therefore finding it necessary to correct their lane position in order to stay within the lane. This may just be an artifact of older drivers taking longer to adapt to the control dynamics of the driving simulator or it may reflect something that is found on the open road. If field studies confirmed that older drivers provided inputs to the steering wheel more often than middle-aged drivers, then this would be consistent with the hypothesis that psychomotor coordination is, at least in part, responsible for the difficulties that older drivers have when treating intersection navigation as a single phase maneuver. In particular, when turning, older drivers will need to make sure that their frequent inputs to the steering wheel do not take them outside their intended path of travel and thus will need to focus continuously on the road ahead of them. This would make it harder to coordinate head and eye movements with turning because a considerable amount of resources is devoted into just steering the vehicle. According to the decoupling hypothesis, the older drivers might have learned to allocate the attentional resources, being progressively more limited with advanced aging, to multiple, concurrent motor responses required for safe intersection maneuver.

There are a number of limitations to note here. First, as with any study done on a driving simulator, the results do not necessarily generalize to the open road. Although close correlations between secondary glance behavior on the simulator and in the field have been reported previously [16], there is no a priori reason to assume that they would apply here. Second, the current size and age characteristics of the sample groups might have led to nonsignificant effects and also limited the nature of the analyses to group-level comparisons (versus correlation approaches). However, despite the relatively small sample size, the experiment yielded sufficient power to detect effects of our interests, and with the other significant, given the direction of each effect, our overall conclusions would not have changed. Additionally, although the two groups in the current study were separated only by 5 years, the statistics [1] and a recent study [33] indicate that it is at age 70 where intersection crash rates begin to rise, and the current results further highlight the criticality of the age in glance and vehicle control behaviors. Third, it is unknown why older drivers might change over time from a two-phase to a one-phase maneuver at intersections, or even whether they do so. Perhaps all drivers of whatever age treat the intersection maneuver as one phase because limited resource of younger drivers can afford concurrent control of multiple responses. Fourth, although it has been shown in a previous study that older drivers can be trained to take secondary glances at an intersection, this study did not show that older drivers in fact treated the intersection maneuver as a two-phase task. The present work merely established that older drivers traveling straight ahead had difficulty in performing concurrent tasks, each requiring different motor responses. But, put together with the results from Romoser and Fisher [16], it indicates why the training would easily produce the results that it did without targeting any particular behavior. Relatedly, future work should also examine a possibility that the training program influences various age-related cognitive declines such as multi-tasking [24], distractability [27], and attentional visual field [28].

From a practical perspective, understanding some of the underlying limitations and outcomes for older drivers in intersections not only helps explain previously observed training effects, but can also be used to enhance and/or develop future interventions. Although the current study focused on secondary glances, it is possible that the approach could be expanded to incorporate more elaborate and individualized patterns of scanning behavior [34]. Furthermore, it is also possible that tests that incorporate the coordination of multiple limbs and eye and head movements (per the decoupling hypothesis)—even outside of driving conditions—could be developed to screen or identify at-risk drivers or those in need of targeted remediation.
